# AS602801 sensitizes glioma cells to temozolomide and vincristine by blocking gap junction communication between glioma cells and astrocytes

**DOI:** 10.1111/jcmm.16375

**Published:** 2021-02-20

**Authors:** Shuai Zhang, Yong Gong, Hongxin Wang, Zhongfan Li, Yunfeng Huang, Xing Fu, Peng Xiang, TianYu Fan

**Affiliations:** ^1^ Department of Neurosurgery ChangSha Central Hospital Changsha China

**Keywords:** AS602801, astrocytes, chemotherapeutic, CX43, glioma, JNK

## Abstract

Previous studies showed that the chemotherapeutic effect of temozolomide (TMZ) and vincristine (VCR) against glioma might be blunted by the co‐culture with astrocytes, and connexin‐43 (CX43) was thought to play a vital role in the communication between glioma cells and astrocytes. In this study, we aimed to investigate the combined chemotherapeutic effect of AS602801 and TMZ/ VCR in glioma cells both. Dye transfer assay was used to evaluate the gap junction activity between U251 cells and astrocytes. Western blot and immunohistochemistry were carried out to analyse the expression of p‐JNK, CX43 and CASP‐3 proteins treated under different conditions. AS602801 significantly suppressed the gap junction activity between U251 cells and astrocytes. The expression of p‐JNK and CX43 was remarkably inhibited by AS602801. TMZ/VCR‐induced apoptosis of glioma cells was effectively enhanced by AS602801 treatment. Accordingly, the inhibitory role of TMZ/VCR in the expression of p‐JNK, CX43 and CASP‐3 in glioma cells was notably restored by AS602801. Furthermore, in a glioma cell xenograft, AS602801 showed an apparent capability to enhance TMZ/VCR‐induced tumour cell apoptosis through altering the expression of p‐JNK, CX43 and CASP‐3. The findings of this study demonstrated that the co‐culture of glioma cells with astrocytes blunted the tumour killing effect of TMZ and VCR. AS602801 down‐regulated CX43 expression by inhibiting JNK. And AS602801 also sensitized glioma cells to TMZ/VCR by blocking the gap junction communication between glioma cells and astrocytes via down‐regulating CX43, indicating its potential role as a novel adjuvant chemotherapeutic agent in the treatment of glioma.

## 1 INTRODUCTION

Gliomas are one of the most deadly intracranial tumours as a result of penetration of tiny tumour in healthy brain tissue that does not allow total resection.^1^ In human brain, persistent inflammation as a result of gliomas leads to the infiltration of microglia as well as astrocytes, while a glial scar containing astrocytes commonly predict gliomas as well as brain metastases.^2^ Although the major function of responsive astrocytes is to shield nerve cells, they likewise up‐regulate the survival of tumour as well as enhance their resistance to chemotherapeutics.^3^ Moreover, responsive astrocytes express several proteins that provide survival advantages to tumour tissues.^4^ Responsive astrocytes have actually been demonstrated to produce interleukin‐6 to assist the growth of tumour tissues and to avoid apoptosis, which helps glioma growth and invasion.^5^ It was actually demonstrated that gliomas were capable to 'make use of' this special function of astrocytes for their survival.^6^

CX43 is one of the most commonly seen connexins in the heart and can easily make up for atrial insufficiency of Cx40 as well as avoid fibrillation, but the replacement of CX43 with various isotypes of Cx increases arrhythmia susceptibility, which might originate from the role of CX43 in regulating various kinases.^7^, ^8^, ^9^ It was actually illustrated that antibodies administered intravenously can bind to CX43 positive cells in glioma, suggesting that the tumour suppressive mechanism of MAbE2 CX43 lies in its suppressing of signalling and/or migration of glioma cells positive for CX43.^10^, ^11^, ^12^ It was shown that gliomas might be resistant to the cytotoxicity induced by TMZ as well as VCR through interacting with astrocytes. This defence mechanism might be actually linked to glioma drug resistance, which also explains the poor outcome of chemotherapeutic treatments with TMZ as well as VCR.^13^

AS602801 is a c‐Jun N‐terminal kinase (JNK) inhibitor and is a unique medication that hinders the tumour initiating ability of cancer stem cells (CSCs).^14^ It was additionally reported that AS602801 possesses the ability to hinder metastasis through the reduction of cell to cell interaction between parenchymal cells and CSCs at metastatic site.^15^ Especially, AS602801 was determined as a prospective medicine for preventing GJ‐dependent interaction of CSCs. It was discovered that AS602801 reduces CX43 expression in CSCs. Most significantly, this medicine will not impair the functionality of molecule transport among astrocytes. It was mentioned that AS602801 certainly will not influence fibroblast IMR90.^14^ As a substance used in endometriosis therapy, AS602801 induces inhibition of JNK and its safety has been validated in clinical trials.^16^, ^17^ AS602801 can be actually considered as an appealing medicine targeting cell communications.^18^, ^19^ Some results demonstrated that AS602801 possesses cytotoxicity against cultured cancer cells as well as primary cancer cells. AS602801 likewise prevented the tumour initiating as well as self‐renewal capability of cancer cells. Most notably, the number of cancer cells in xenograft tumours is actually decreased by 40 mg/kg of AS602801 without impacting the wellness of tumour‐bearing rats.^14^

It has been shown that the chemotherapeutic effect of TMZ and VCR against glioma might be blunted by the co‐culture with astrocytes, and CX43 was thought to play a vital role in the communication between glioma cells and astrocytes.^13^, ^20^, ^21^ Specially, glioma‐associated NHAs were reported to protect MGMT‐negative glioma cells from TMZ‐induced apoptosis by the functional intercellular transfer of exosomal MGMT mRNA.^20^ Furthermore, AS602801, an inhibitor or JNK activation, was shown to suppress the expression of CX43.^15^ In this study, we tested the chemotherapeutic effect of AS602801 together with TMZ or VCR in killing glioma cells both in vitro and in vivo.

## 2 MATERIALS AND METHODS

### 2.1 Materials

AS602801 was bought from AdooQ Bio Science and then dissolved in DMSO to make a 10 mmol/L stock solution.

### 2.2 Animal and treatment

The animal experiment in this study was approved based on the Rule of Animal Management issued by the Ministry of Health in China. For the formation of subcutaneous xenografts, 1 × 10^7^ of glioma cells were injected in to the legs of 6 week old NOD/SCID mice bought from the Chinese Academy of Medical Science. When the size of subcutaneous tumour was 50 mm^3^ (D10 after the subcutaneous implant of tumour cells), 10 mice with an identical tumour volume were randomly assigned into 3 groups, that is 1. GLIOMA group (mice implanted with glioma cells and received no treatment); 2. GLIOMA + TMZ group (mice implanted with glioma cells and received treatment with 50 mg/kg of TMZ); and 3. GLIOMA + TMZ + AS 602801 group (mice implanted with glioma cells and received treatment with 50 mg/kg of TMZ plus 20 mg/kg of AS 602801). For the AS602801 treatment, AS602801 was given through gavage once a day for 30 consecutive days. To study the effect of VCR, a similar number of mice were also divided into 3 other groups with 10 mice in each group, that is 1. GLIOMA group (mice implanted with glioma cells and received no treatment); 2. GLIOMA + VCR group (mice implanted with glioma cells and received treatment with 50 mg/kg of VCR); and 3. GLIOMA + VCR + AS 602801 group (mice implanted with glioma cells and received treatment with 50 mg/kg of VCR plus 20 mg/kg of AS 602801). The AS602801 treatment for the mice in the GLIOMA + VCR +AS 602801 group was the same as that in the GLIOMA + TMZ + AS 602801 group, that is AS602801 was given through gavage once a day for 30 consecutive days. At D35 after the mice were implanted with subcutaneous injection of glioma cells, the mice were killed via cervical dislocation to measure the width (W) as well as length (L) of each tumour by using calipers, so that tumour volume in each mouse could be computed as (L × W2) × 0.5. In addition, tumour tissues were collected from each mouse for the further analysis. The institutional ethical committee has approved the protocol of this study.

### 2.3 Cell culture and transfection

U251 cells and astrocytes used in this particular research were cultured in tissue culture dishes coated with collagen type I (IWAKI) in a DMEM/F‐12 medium (Gibco, Thermo Fisher Scientific) supplemented by 1% B27 (Thermo Fisher Scientific), 25 ng/mL of EGF, 25 ng/mL of FGF‐2 (Peprotech), 26 mmol/L of d‐(+)‐glucose, 4.5 mmol/L of l‐glutamine, 100 U/mL penicillin and 100 μg/mL of streptomycin (Gibco, Thermo Fisher Scientific). The culture conditions were 37°C, 90% humidity and 5% CO_2_. Primary mouse astrocytes were separated from cerebral cortex of newborn mice by using a 70 μm cell filter. Then, primary mouse astrocytes were cultured separately first in a DMEM medium added with 100 μg/mL streptomycin, 100 unit/mL penicillin and 10% FBS.

### 2.4 Co‐culture experiment of chemo resistance

To determine whether astrocytes can shield glioma cells against apoptosis caused by chemotherapeutics, we made use of an in vitro co‐culture system of astrocyte‐glioma. In brief, U251 cells and astrocytes were divided into several sets of groups shown below.

In the first set of groups, U251 cells were divided into 2 groups, that is 1. Control group (U251 cells treated with PBS) and 2. AS602801 group (U251 cells treated with 10 mmol/L of AS602801^15^). The cells in both groups were harvested after 72 hours of treatment for subsequent analyses.

In the second set of groups, GLIOMA cells were divided into 4 groups, that is 1. GLIOMA group (GLIOMA cells treated with PBS); 2. GLIOMA + TMZ group (GLIOMA cells treated with 5 μmol/L of TMZ^6^); 3. GLIOMA + TMZ + NHA group (GLIOMA cells treated with 5 μmol//L of TMZ and co‐cultured with NHA; NHA: nanoscale hydroxyapatite); and 4. GLIOMA + TMZ + NHA + AS602801 group (GLIOMA cells treated with 5 μmol/L of TMZ, co‐cultured with NHA and treated with 10 mmol/L of AS602801).

In the third set of groups, GLIOMA cells were divided into four groups, that is 1. GLIOMA group (GLIOMA cells treated with PBS); 2. GLIOMA + VCR group (GLIOMA cells treated with 4nM of VCR); 3. GLIOMA + VCR + NHA group (GLIOMA cells treated with 4 nmol/L of VCR and co‐cultured with NHA); and 4. GLIOMA + VCR + NHA + AS602801 group (GLIOMA cells treated with 4 nmol/L of VCR, co‐cultured with NHA and treated with 10 mmol/L of AS602801).

The cells in the second and third set of groups were all harvested after 72 hours of treatment for subsequent analyses. In the co‐culture experiments, glioma cells were first cultured alone/co‐cultured along with NHA in a 2:1 proportion. Twenty‐four hours later, glioma cells were treated with 1000 μmol/L of TMZ or 4 nmol/L of VCR depending on the experimental grouping.^13^ At 72 hours of culture, the cells in all groups were stained along with Annexin V‐APC as well as propidium iodide to evaluate the apoptosis status of CFSE positive glioma cells via flow cytometry or other analyses. To find out whether chemo resistance of glioma cells was because of interaction between glioma cells and astrocytes themselves or just particular cytokines produced by astrocytes, a transwell culture was utilized by plating NHA to the transwell chamber located on the top while seeding the parental generation of culture cells to the transwell chamber located at the bottom. After the chemotherapeutics were added for 48 hours, the apoptotic portion of cells was assayed by using flow cytometry.

### 2.5 Dye transfer assay

A dye transfer assay was performed to evaluate the effect of AS602801 on the cell‐cell communication between U251 and astrocytes. In brief, donor cells were tagged for 30 minutes by using 1 mg/mL of a calcein‐AM dye (Nacalai) according to the experimental protocol provided by the manufacturer, while the acceptor cells were tagged by using 10 μmol/L of a DiD dye (Takara). The donor cells were then co‐cultured in a 1:1 proportion along with the acceptor cells for 6 hours free of chemotherapeutics before the evaluation of calcein transfer to the acceptor cells from donor cells was done by making use of a FACS Canto II Flow Cytometer (BD) according to the experimental protocol provided by the manufacturer. Data were analysed by making use of the FlowJo software (Treestar).

### 2.6 MTT cell proliferation assay

The status of cell proliferation was evaluated by utilizing an MTT assay (Thermo Fisher Scientific) according to the experimental protocol provided by the manufacturer. The absorbance was determined at a 450 nm wavelength.

### 2.7 Western blot analysis

Cell and tissue samples were lysed in a RIPA buffer (Thermo Fisher Scientific) according to the experimental protocol provided by the manufacturer to isolate proteins, which were then resolved using a 10% SDS‐PAGE gel and blotted onto polyvinylidene difluoride membranes, which were then blocked using 0.1% BSA and treated with anti‐p‐JNK, anti‐CX43 and anti‐CASP‐3 primary antibodies as well as suitable HRP‐tagged secondary antibodies in sequence using conditions provided by the antibody manufacturer (Abcam). Finally, the relative protein expression of p‐JNK, CX43 and CASP‐3 in each sample was analysed by utilizing the Image J software.

### 2.8 Apoptosis analysis

Glioma cells were treated with TMZ, TMZ + NHA and TMZ + NHA + AS602801 before flow cytometry was performed to evaluate the apoptosis of glioma cells treated under different treatments. The status of cell apoptosis was evaluated by using an Annexin V‐FITC and PI assay kit (Thermo Fisher Scientific) according to the experimental protocol provided by the manufacturer on a FACS Canto II Flow Cytometer (BD).

### 2.9 Immunohistochemistry

Paraffin‐embedded tissue sections were de‐waxed as well as rehydrated before they were subject to antigen retrieval. Then, the sections were blocked with BSA, incubated with primary and secondary anti‐CX43 antibodies (Abcam), developed by using a VECTASTAIN Elite ABC assay kit (Vector Laboratories) according to the experimental protocol provided by the manufacturer and counter stained with DAPI before the expression of CX43 was evaluated.

### 2.10 TUNEL assay

The apoptosis of tissue samples was evaluated by using a Terminal deoxynucleotidyl transferase (TdT) dUTP Nick‐End Labeling (TUNEL) assay (Wako Pure Chemical) according to the experimental protocol provided by the manufacturer.

### 2.11 Statistical analysis

All results were shown as mean ± standard deviations. All statistical analyses were done in SPSS 19.0 (SPSS). Student's *t* test and one‐way ANOVA were used for statistical comparison, and Tukey's test was used as a post hoc test for one‐way ANOVA *P* < .05 was taken into consideration as statistically significant.

## 3 RESULTS

### 3.1 AS602801 suppressed the gap junction activity between U251 cells and astrocytes

**FIGURE 1:**
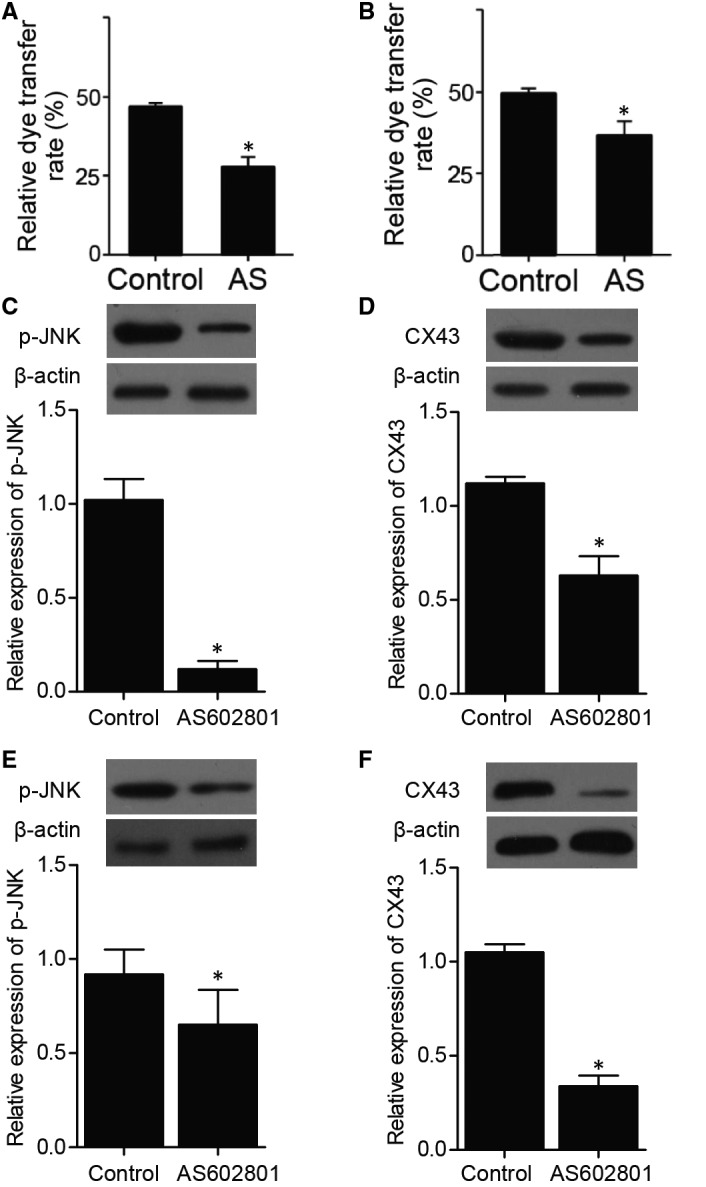
AS602801 suppressed the gap junction activity between U251 cells and astrocytes as well as the expression of p‐JNK and CX43. A, Quantification of dye transfer from U251 cells to astrocytes showed that AS602801 suppressed the dye transfer from U251 cells to astrocytes (*P‐value < .05 compared with control; Student's *t* test). B, Quantification of dye transfer from astrocytes to U251 cells showed that AS602801 suppressed the dye transfer from astrocytes to U251 cells (*P‐value < .05 compared with control; Student's *t* test). C, Western blot analysis showed that p‐JNK protein expression was attenuated in U251 cells (*P‐value < .05 compared with control; Student's *t* test). D, Western blot analysis showed that CX43 protein expression was attenuated in U251 cells (*P‐value < .05 compared with control; Student's *t* test). E, Western blot analysis showed that p‐JNK protein expression was attenuated in astrocytes (*P‐value < .05 compared with control; Student's *t* test). F, Western blot analysis showed that CX43 protein expression was attenuated in astrocytes (*P‐value < .05 compared with control; Student's *t* test)

A dye transfer assay was performed to evaluate the effect of AS602801 on the cell‐cell communication between U251 cells and astrocytes. U251 cells and astrocytes were labelled with calcein as donor cells, respectively, and the transfer of calcein to acceptor cells was quantified by flow cytometry. The percentage of acceptor cells with calcein fluorescence was significantly decreased by AS602801 treatment (Figure 1A,B), indicating that the gap junction activity between U251 cells and astrocytes was remarkably suppressed by AS602801. Furthermore, Western blot was carried out to assess the expression of p‐JNK and CX43. The expression of p‐JNK and CX43 was remarkably suppressed by AS602801 under different circumstances (Figure 1C‐F).

### 3.2 AS602801 rescued NHA‐abolished suppression of glioma cell proliferation by TMZ and VCR

**FIGURE 2:**
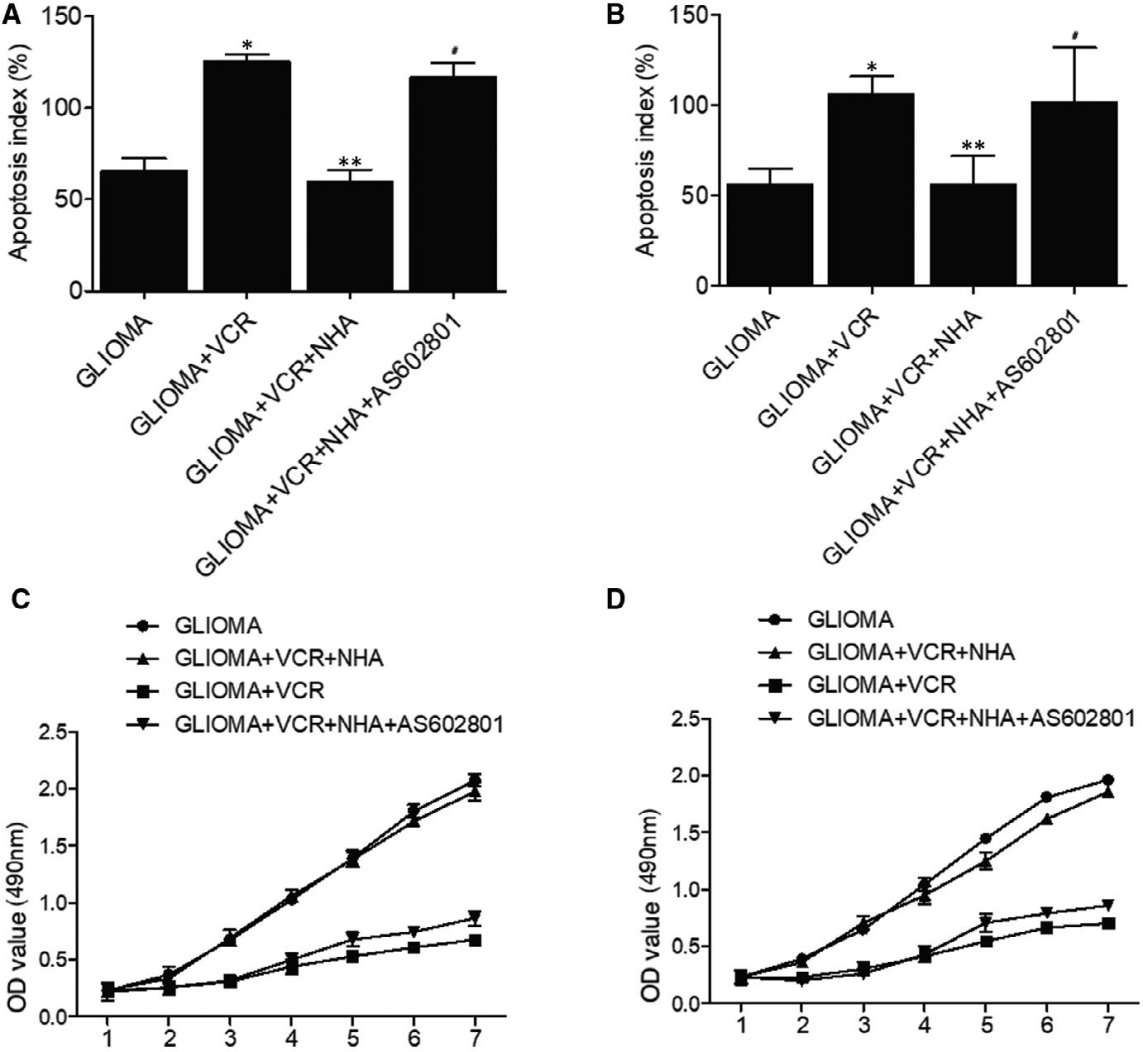
AS602801 restored NHA‐abolished therapeutic role of TMZ and VCR in glioma cells. A, Flow cytometry analysis showed that TMZ‐induced apoptosis, which was abolished by NHA, was maintained by AS602801 (*P‐value < .05 compared with glioma group; **P‐value < .05 compared with glioma + VCR group; ^#^ *P*‐value < .05 compared with glioma + VCR + NHA group; one‐way ANOVA and Tukey's test). B, Flow cytometry analysis showed that VCR‐induced apoptosis, which was abolished by NHA, was restored by AS602801 (*P‐value < .05 compared with glioma group; **P‐value < .05 compared with glioma + VCR group; ^#^ *P*‐value < .05 compared with glioma + VCR + NHA group; one‐way ANOVA and Tukey's test). C, MTT assay showed that TMZ‐induced decrease in proliferation, which was abolished by NHA, was restored by AS602801. D, MTT assay showed that VCR‐induced decrease in proliferation, which was abolished by NHA, was restored by AS602801

The apoptosis of glioma cells was remarkably elevated with TMZ and TMZ + NHA + AS602801 treatments, whereas no obvious difference was observed for glioma cells treated with TMZ + NHA (Figure 2A). These results demonstrated that NHA abolished the therapeutic effect of TMZ on glioma cells, and the effect was restored by AS602801 treatment. Similarly, AS602801 treatment showed evident effects on restoring NHA‐abolished therapeutic effect of VCR treatment on glioma cells (Figure 2B). Moreover, MTT assay was used to evaluate the proliferation of glioma cells treated under different conditions. NHA treatment abolished TMZ/VCR‐induced suppression of glioma cell proliferation, which was restored by AS602801 treatment (Figure 2C,D).

### 3.3 AS602801 treatment activated TMZ and VCR‐induced suppression of p‐JNK, CX43 and CASP‐3 protein expression in glioma cells

**FIGURE 3:**
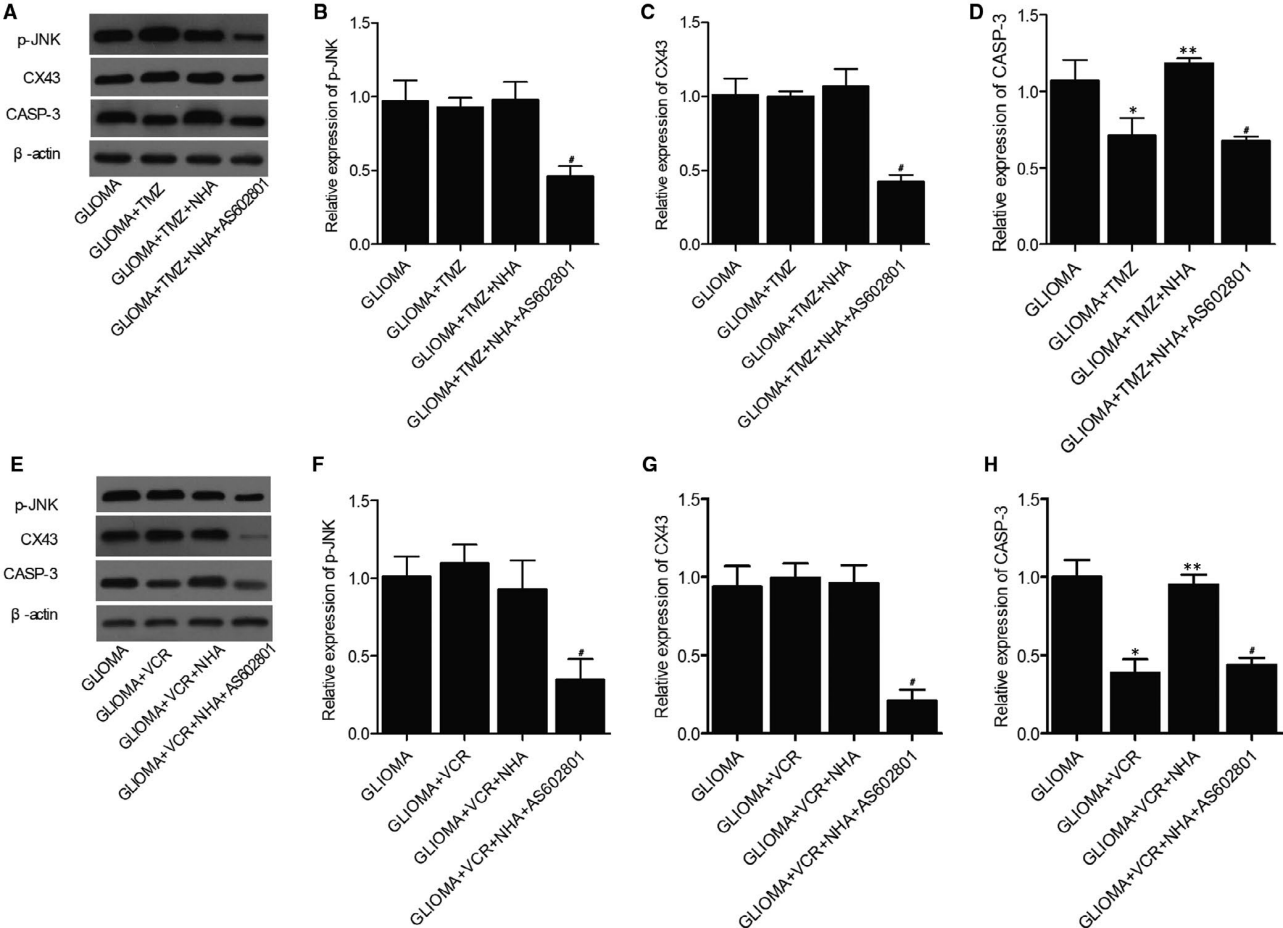
AS602801 restored NHA‐abolished suppressive role of TMZ and VCR in p‐JNK/CX43/CASP‐3 protein expression in glioma cells. A, Western blot analysis showed that AS602801 suppressed the expression of p‐JNK/CX43/CASP‐3 proteins in TMZ‐treated glioma cells. B, Quantification of p‐JNK protein expression showed that AS602801 suppressed the expression of p‐JNK protein in TMZ‐treated glioma cells (^#^ *P*‐value < .05 compared with glioma + TMZ + NHA group; one‐way ANOVA and Tukey's test). C, Quantification of CX43 protein expression showed that AS602801 suppressed the expression of p‐JNK protein in TMZ‐treated glioma cells (^#^ *P*‐value < .05 compared with glioma + TMZ + NHA group; one‐way ANOVA and Tukey's test). D, Quantification of CASP‐3 protein expression showed that AS602801 suppressed the expression of p‐JNK protein in TMZ‐treated glioma cells (*P‐value < .05 compared with glioma group; **P‐value < .05 compared with glioma + TMZ group; ^#^ *P*‐value < .05 compared with glioma + TMZ + NHA group; one‐way ANOVA and Tukey's test). E, Western blot analysis showed that AS602801 suppressed the expression of p‐JNK/CX43/CASP‐3 proteins in VCR‐treated glioma cells. F, Quantification of p‐JNK protein expression showed that AS602801 suppressed the expression of p‐JNK protein in VCR‐treated glioma cells (^#^ *P*‐value < .05 compared with glioma + VCR + NHA group; one‐way ANOVA and Tukey's test). G, Quantification of CX43 protein expression showed that AS602801 suppressed the expression of p‐JNK protein in VCR‐treated glioma cells (^#^ *P*‐value < .05 compared with glioma + VCR + NHA group; one‐way ANOVA and Tukey's test). H, Quantification of CASP‐3 protein expression showed that AS602801 suppressed the expression of p‐JNK protein in VCR‐treated glioma cells (*P‐value < .05 compared with glioma group; **P‐value < .05 compared with glioma + VCR group; ^#^ *P*‐value < .05 compared with glioma + VCR + NHA group; one‐way ANOVA and Tukey's test)

Next, Western blot was used to assess the different expression of p‐JNK, CX43 and CASP‐3 in glioma cells treated under different conditions. No obvious difference was observed for the expression of p‐JNK (Figure 3A,B,E,F) and CX43 (Figure 3A,C,E,G) in glioma cells treated with TMZ/VCR alone and in a combined manner with NHA, but p‐JNK and CX43 expression was significantly repressed in glioma cells treated with TMZ + NHA + AS602801 and VCR + NHA + AS602801. However, CASP‐3 expression was decreased in glioma cells treated with TMZ/VCR alone. NHA treatment reversed TMZ/VCR‐induced suppression of CASP‐3 treatment, whereas AS602801 restored the effect of TMZ/VCR alone on CASP‐3 suppression (Figure 3A,D,E,H).

### 3.4 AS602801 restored NHA‐abolished glioma cell apoptosis induced by TMZ and VCR

**FIGURE 4:**
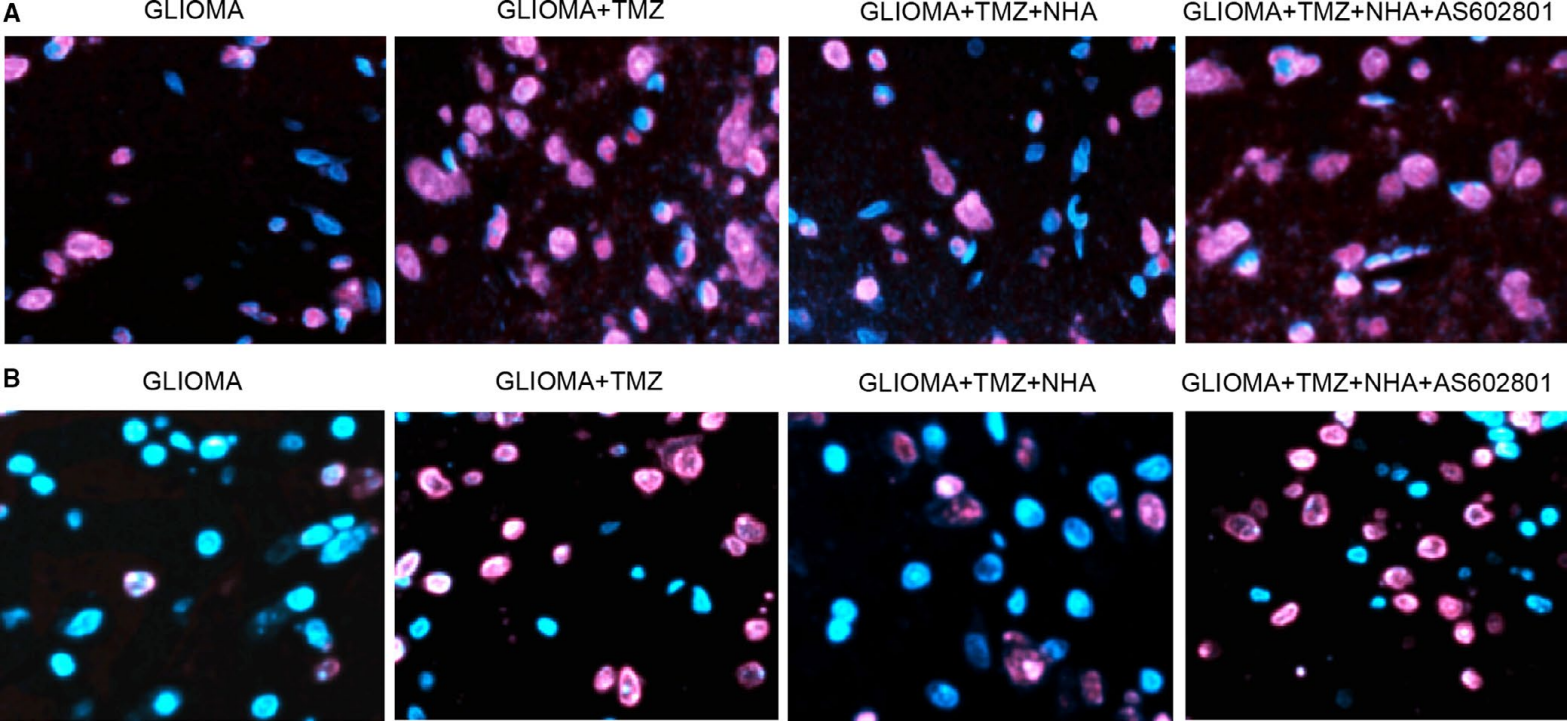
AS602801 restored NHA‐abolished apoptosis of glioma cells. A, TMZ‐induced apoptosis of glioma cells, which was abolished by NHA, was restored by AS602801. B, VCR‐induced apoptosis of glioma cells, which was abolished by NHA, was restored by AS602801

Fluorescence assay was further adopted to measure the apoptosis of glioma cells treated under different conditions. TMZ (Figure 4A) and VCR (Figure 4B) treatments alone significantly elevated the apoptosis of glioma cells, which was abolished by NHA. Further treatment with AS602801 notably re‐activated the apoptosis of glioma cells induced by TMZ and VCR.

### 3.5 AS602801 sensitized TMZ/VCR‐induced down‐regulation of p‐JNK, CX43 and CASP‐3 expression in glioma cell xenograft in nude mice

**FIGURE 5:**
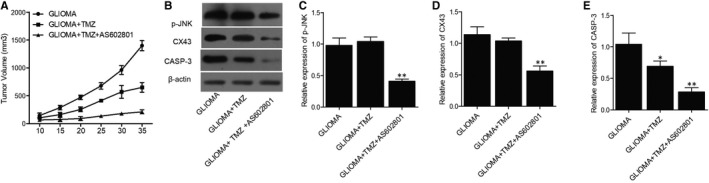
AS602801 activated the suppressive role of TMZ in the growth of xenograft and altered the expression of p‐JNK, CX43 and CASP‐3. A, The growth of glioma cell xenograft in nude mice was inhibited by TMZ treatment and the inhibitory effect was further enhanced by AS602801. B, Western blot showed different expression of p‐JNK, CX43 and CASP‐3 proteins in glioma cell xenograft treated with different therapeutic strategies. C, Quantification of p‐JNK protein expression showed that p‐JNK expression was repressed by TMZ + AS602801 (**P‐value < .05 compared with glioma + TMZ group; one‐way ANOVA and Tukey's test). D, Quantification of CX43 protein expression showed that CX43 expression was repressed by TMZ + AS602801 (**P‐value < .05 compared with glioma + TMZ group; one‐way ANOVA and Tukey's test). E, Quantification of CASP‐3 protein expression showed that CX43 expression was repressed by TMZ and the inhibitory effect was further enhanced by AS602801 (*P‐value < .05 compared with glioma group; **P‐value < .05 compared with glioma + TMZ group; one‐way ANOVA and Tukey's test)

**FIGURE 6:**
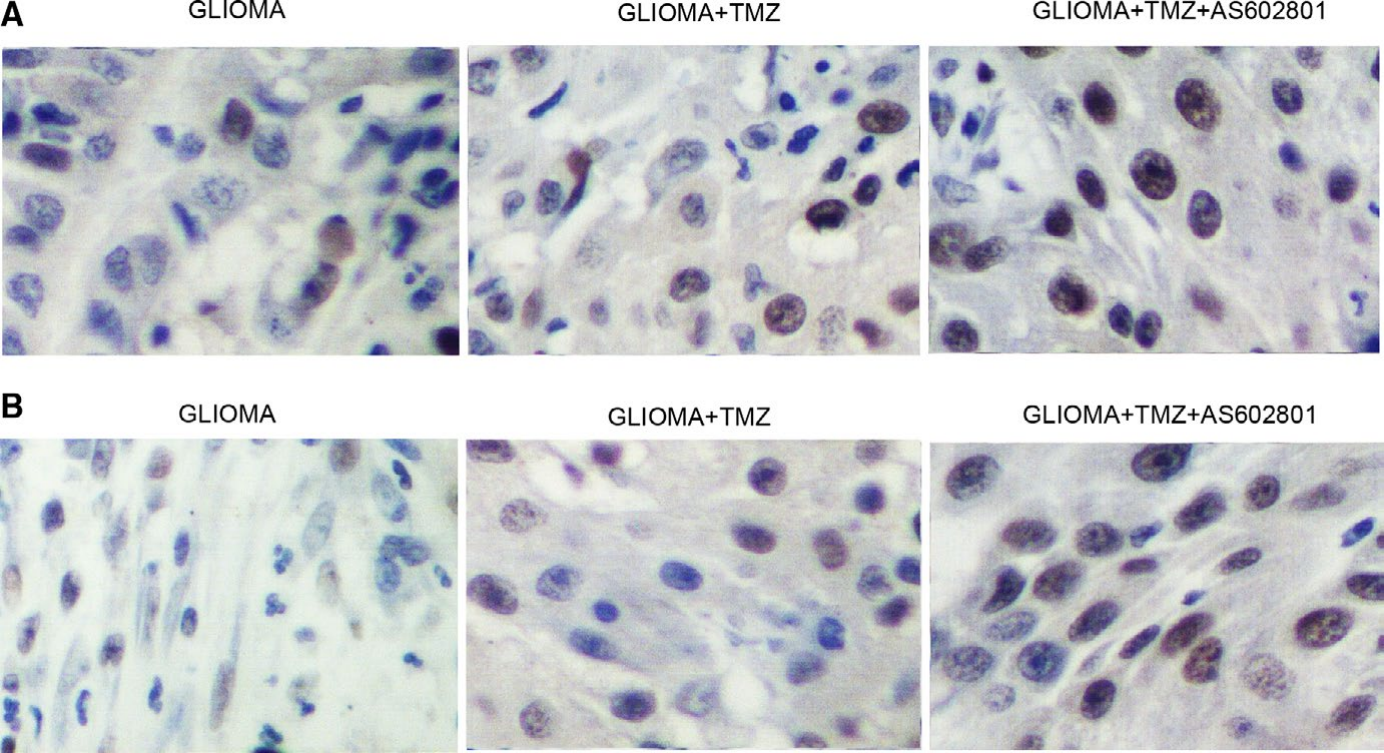
AS602801 activated the suppressive role of TMZ in CX43 expression and apoptosis of glioma cell xenograft in nude mice. A, IHC analysis showed that CX43 protein expression was repressed by TMZ + AS602801 in glioma cell xenograft in nude mice. B, TUNEL analysis showed that AS602801 activated the suppressive role of TMZ in the apoptosis of glioma cell xenograft in nude mice

**FIGURE 7:**
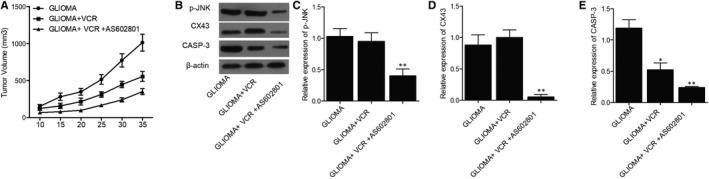
AS602801 activated the suppressive role of VCR in the growth of xenograft and altered the expression of p‐JNK, CX43 and CASP‐3. A, The growth of glioma cell xenograft in nude mice was inhibited by VCR treatment and the inhibitory effect was further enhanced by AS602801. B, Western blot showed different expression of p‐JNK, CX43 and CASP‐3 proteins in glioma cell xenograft treated with different therapeutic strategies. C, Quantification of p‐JNK protein expression showed that p‐JNK expression was repressed by VCR + AS602801 (**P‐value < .05 compared with glioma + VCR group; one‐way ANOVA and Tukey's test). D, Quantification of CX43 protein expression showed that CX43 expression was repressed by VCR + AS602801 (**P‐value < .05 compared with glioma + TMZ group; one‐way ANOVA and Tukey's test). E, Quantification of CASP‐3 protein expression showed that CX43 expression was repressed by VCR and the inhibitory effect was further enhanced by AS602801 (*P‐value < .05 compared with glioma group; **P‐value < .05 compared with glioma + VCR group; one‐way ANOVA and Tukey's test)

**FIGURE 8:**
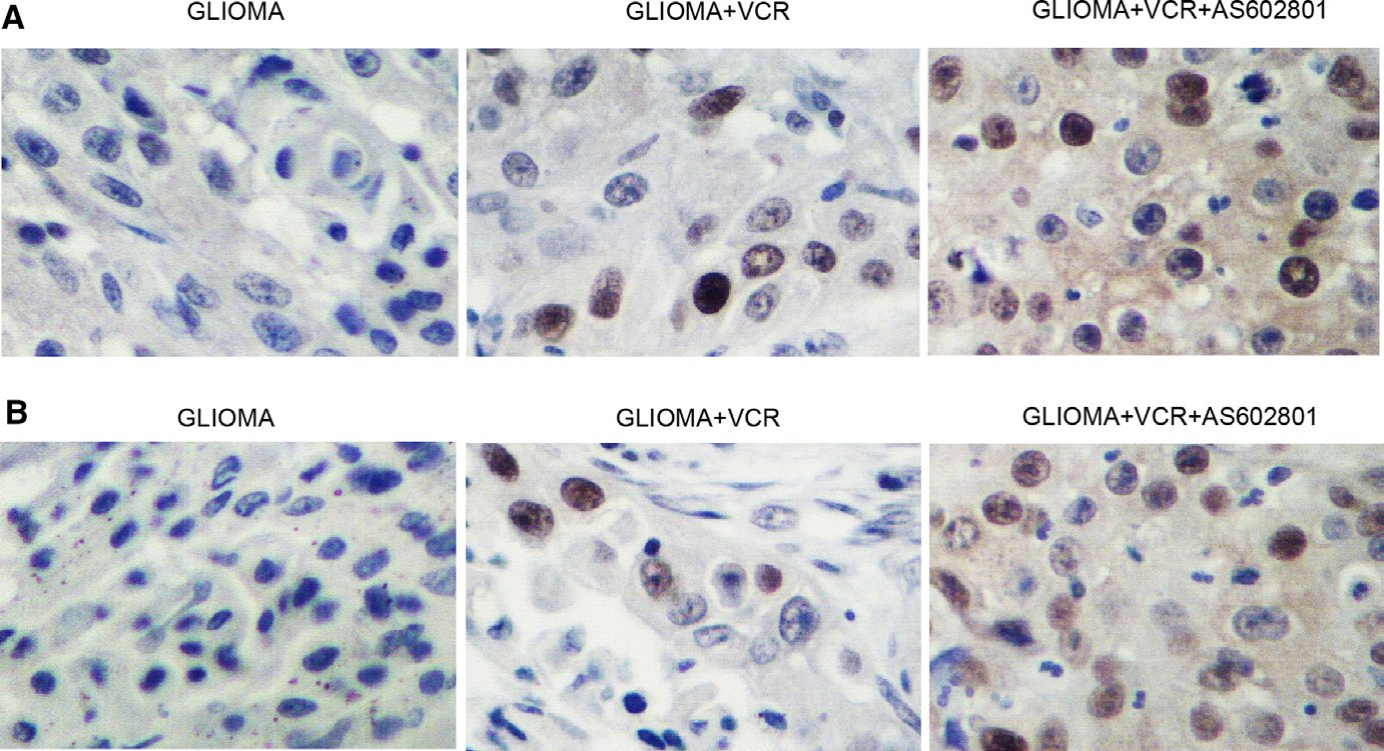
AS602801 activated the suppressive role of VCR in CX43 expression and apoptosis of glioma cell xenograft in nude mice. A, IHC analysis showed that CX43 protein expression was repressed by VCR + AS602801 in glioma cell xenograft in nude mice. B, TUNEL analysis showed that AS602801 activated the suppressive role of VCR in the apoptosis of glioma cell xenograft in nude mice

Glioma cell xenograft was planted in nude mice, which were then treated with TMZ alone or in a combined treatment with AS602801. We found that the tumour volume was remarkably suppressed by TMZ alone and more evidently inhibited by TMZ + AS602801 (Figure 5A). Western blot analysis showed that TMZ treatment showed no effect on the expression of p‐JNK and CX43, but the combined treatment with AS602801 remarkably diminished the expression of p‐JNK (Figure 5B,C) and CX43 (Figure 5B,D). However, TMZ treatment alone and combined treatment with AS602801 progressively activated the expression of CASP‐3 in nude mice carrying the glioma cell xenograft (Figure 5B,E). Furthermore, immunohistochemistry analysis confirmed the effect of AS602801 on sensitizing CX43 to the inhibitory effect of TMZ (Figure 6A), and TUNEL analysis showed that the apoptosis in glioma cell xenograft was progressively decreased by TMZ administration alone and in a combined treatment with AS601801 (Figure 6B). Meanwhile, the tumour volume of glioma cell xenograft in nude mice was remarkably suppressed by VCR alone and more evidently inhibited by TMZ + AS602801 (Figure 7A). AS602801 + VCR declined the expression of p‐JNK (Figure 7B,C) and CX43 (Figures 7B,D,8A) proteins in glioma cell xenograft, whereas the expression of CASP‐3 was increased by the AS602801 + VCR treatment (Figure 7B,E). The apoptosis in glioma cell xenograft was enhanced by the treatment with VCR + AS601801 (Figure 8B).

## 4 DISCUSSION

The microenvironment of organs has actually been related to the progression as well as survival of tumour.^22^ Astrocytes are actually the most dominant type of cells in human brain by connecting one another via means of gap junctional communication (GJC).^23^ Astrocytes also act as house‐keeping cells to preserve homeostasis in the microenvironment of brain.^24^, ^25^ In medical disorders, astrocytes are triggered by means of the up‐regulation of glial fibrillary acid proteins (GFAP) to guard nerve cells against numerous neuron damages, injuries and ischaemic insult.^26^, ^27^ Previous research demonstrated a possibility that astrocytes in glioma can also guard glioma cells against the cytotoxicity of chemotherapeutics in vivo.^28^, ^29^ AS602801 is actually a JNK inhibitor developed to deal with neurological conditions.^16^ The effectiveness of AS602801 in endometriosis treatment has actually been verified.^30^, ^31^ It was illustrated that AS602801 can inhibit CSCs both in vivo and in vitro.^14^ AS602801 especially decreased cell‐cell interaction of A549 CSCs with no reductions of GJ communication among astrocytes, making AS602801 a prospective medicine in the treatment of brain metastasis.^15^ In this study, we implanted a glioma xenograft in nude mice and treated the mice with TMZ or VCR alone and in a combined treatment with AS602801. AS602801 activated the suppressive effect of TMZ/VCR on tumour growth in nude mice and also enhanced the inhibitory role of TMZ/VCR in p‐JNK/CX43 expression and their promotive effect on CASP‐3 expression. In addition, we carried out IHC and TUNEL assays to analyse the expression of CX43 and apoptosis of glioma cell xenograft treated under different conditions. AS602801 effectively attenuated the expression of CX43 and diminished the apoptosis of the xenograft.

It was actually shown that through decreasing CX43 expression with siRNA in astrocytes, these astrocytes, which are usually resistant to H2O2‐induced apoptosis, can become sensitized, suggesting that the anti‐apoptotic ability of CX43 also appears in usual astrocytes.^32^ A lot of mechanisms have actually been suggested for CX43‐induced cell migration, yet the effect of homocellular GJIC on glioma infiltration is still not thoroughly studied.^33^, ^34^, ^35^ It has actually been revealed that CX43 in astrocytes plays a significant role in regulating glioma invasion via stromal cells.^36^, ^37^

CX43 is a gap junction protein and is commonly found in mature astrocytes.^38^ CX43 expression is enhanced in responsive astrocytes in several pathologies of brain.^39^, ^40^ CX43 belongs to the family of intercellular channel proteins that enable the transport of metabolites like sugar among adjacent cells.^41^, ^42^ Moreover, gap junction communication plays a notable role in calcium signalling in cells.^43^, ^44^ However, unpaired CX43 hemichannel opens under pathological conditions to cause the secretion of molecules consisting of ATP, glutamate and calcium ions from cells.^45^, ^46^ Research up until now has considered CX43 as a negative regulator of growth of glioma cells.^47^ It seems that CX43 influences cell cycles through blocking the entry to the S or M phase, indicating a decline in the levels of proteins associated with cell cycle progression, such as cyclins A, D2, D1, cdk5, E, skp2 and cdk6.^48^, ^49^, ^50^ In this study, we performed dye transfer assay to evaluate the gap junction activity between U251 cells and astrocytes treated AS602801. AS602801 significantly suppressed the gap junction activity between U251 cells and astrocytes. Furthermore, we carried out flow cytometry, MTT assay and fluorescence assay to measure the apoptosis and proliferation of glioma cells treated with TMZ/VCR alone or in combined treatment with NHA and AS602801. AS602801 effectively restored the therapeutic effect of TMZ and VCR on glioma cells.

Reports linking cell migration to connexins showed that such association was first evidenced by neural crest cell migration regulated by CX43 expression.^51^ Such cells display a reduced mobility when is silenced, while CX43 overexpression boosts cell motility.^52^, ^53^ It has actually been revealed that injury‐induced apoptosis was elevated in newborn mice carrying null‐mutant CX43.^54^ Moreover, inhibitors of gap junction, including carbenoxolone as well as 18‐beta‐glycyrrhetinic acid, can protect cells against apoptosis.^55^, ^56^ While the mechanism underlying the apoptotic effect of CX43 remains unclear, it may be associated with anti‐apoptotic molecule bcl‐2.^57^ In this study, we performed Western blot to assess the expression of p‐JNK, CX43 and CASP‐3 in glioma cells treated under different conditions. Considering the report that enhancement of CX43‐based gap junctional communication promoted chemoresistance to TMZ and VCR of cancer cells^6^ and AS602801 down‐regulated expression of CX43 via modulating activation of JNK^15^ together with the data obtained from this study, AS602801 sensitized glioma cells to the inhibitory effect of TMZ and VCR via regulating signalling pathway of p‐JNK/CX43/CASP‐3 expression.

## 5 CONCLUSION

The findings of this study demonstrated that the co‐culture of glioma cells with astrocytes blunted the tumour killing effect of TMZ and VCR. AS602801, an inhibitor of JNK, down‐regulated the expression of CX43 by inhibiting the expression of JNK. Furthermore, the administration of AS602801 sensitizes glioma cells to TMZ and VCR by blocking the gap junction communication between glioma cells and astrocytes via down‐regulating connexin‐43 expression. Thus, AS602801 might a used as a novel adjuvant chemotherapeutic agent in the treatment of glioma.

## CONFLICT OF INTEREST

None.

## AUTHOR CONTRIBUTIONS

**Shuai Zhang:** Conceptualization (equal); Investigation (equal); Resources (equal); Software (equal); Supervision (equal); Writing‐original draft (equal). **Yong Gong:** Conceptualization (equal); Funding acquisition (equal); Investigation (equal); Methodology (equal); Project administration (equal); Writing‐original draft (equal). **Hongxin Wang:** Investigation (equal); Methodology (equal); Validation (equal); Visualization (equal). **Zhongfan Li:** Validation (equal); Writing‐original draft (equal). **Yunfeng Huang:** Formal analysis (equal); Investigation (equal); Software (equal); Validation (equal). **Xing Fu:** Formal analysis (equal); Investigation (equal). **Peng Xiang:** Conceptualization (equal); Investigation (equal); Validation (equal). **TianYu Fan:** Funding acquisition (equal); Methodology (equal); Project administration (equal); Resources (equal); Supervision (equal); Writing‐original draft (equal).

## Data Availability

The data that support the findings of this study are available from the corresponding author upon reasonable request.
